# Unraveling the Myths Around Epilepsy: A Cross-Sectional Study of Knowledge, Attitude, and Practices Among Pakistani Individuals

**DOI:** 10.7759/cureus.39760

**Published:** 2023-05-31

**Authors:** Tahleel Javed, Hasan A Awan, Nahl Shahzad, Deewan Ojla, Hanniya B Naqvi, Hafsah Arshad, Syeda B Owais, Shazil Abrar

**Affiliations:** 1 Department of Neuropsychiatry, St Matthews Healthcare, Northampton, GBR; 2 Department of Neuropsychiatry, South London and Maudsley NHS Foundation Trust, London, GBR; 3 Department of Medicine, Nottingham University Hospitals NHS Trust, Nottingham, GBR; 4 Department of Medicine, West Hertfordshire Teaching Hospitals NHS Trust, Watford, GBR; 5 Department of Medicine, Fauji Foundation Hospital, Rawalpindi, PAK; 6 Department of Medicine, Combined Military Hospital, Rawalpindi, PAK; 7 Department of Neurology, Fauji Foundation Hospital, Rawalpindi, PAK

**Keywords:** public education, stigma, awareness, misconceptions, myths, pakistan, practices, attitude, knowledge, epilepsy

## Abstract

Introduction: Across its historical trajectory, epilepsy has frequently been linked to evil forces, particularly in the sub-continent. This research was created to find out if educated Pakistanis still believe that epilepsy is caused by being possessed by spirits (Jinns). The objective of the study is to assess the knowledge, attitudes, and practices (KAP) regarding epilepsy within the educated populace of Pakistan.

Method: After approval from the Ethical Review Committee, a population-based cross-sectional design was conducted in Chakwal District, Pakistan between February 1, 2018, and June 1, 2020, to evaluate the general knowledge and attitudes of the public toward epilepsy. A non-probability convenience sampling technique was utilized to recruit participants from different socioeconomic backgrounds across Chakwal District, and only individuals aged 18 years or older with at least 12 years of education were eligible to participate. A previously validated structured questionnaire was used to document findings. The study focused on several variables, such as knowledge about epilepsy and the percentage of people who have witnessed seizures, as well as sources of knowledge, subjective causes of epilepsy, beliefs in cure, transmission, and treatment options.

Results: The survey included 512 participants, and the age distribution was as follows: 18-29 years old accounted for 18% of the respondents, 30-44 years old accounted for 35%, and 45-60 years old accounted for 31%. There was a female predominance with a frequency of 312 (60.9%). When asked about their sources of knowledge about epilepsy, the majority of participants (59.57%) reported learning about epilepsy from friends and relatives. A smaller percentage (18.36%) reported learning about epilepsy from schools, while another 20.31% heard about epilepsy from media and relatives.

Conclusion: The results of this research show that the general populace of Pakistan has a serious dearth of comprehension and information about epilepsy. Participants frequently held misconceptions about epilepsy being a hereditary disease and a mental condition, highlighting the need for focused education and information efforts to dispel these falsehoods. The fact that most participants got their knowledge about epilepsy from peers and family also emphasizes the value of peer education and social networks in spreading awareness of the disease.

## Introduction

Epilepsy is a neurological disorder that impacts an estimated 50 million individuals globally, with four-fifth of the cases reported in low- and middle-income countries [1]. Epilepsy is characterized by recurrent seizures, which are sudden and uncontrolled surges of electrical activity in the brain. Despite significant advances in understanding epilepsy and its treatment, people with epilepsy continue to face stigma, discrimination, and social exclusion, leading to a reduced quality of life [2].

Epilepsy is a neurological condition that impacts people of all ages and socioeconomic groups. It manifests in the form of recurrent seizures, which can vary in severity and frequency. Epilepsy continues to be a considerable public health issue globally, especially in low- and middle-income nations, despite substantial improvements in its detection and management [3]. The proportion of people with epilepsy who do not receive the proper care is known as the treatment gap, and in some areas, that number is as high as 75% [4]. In Nigeria, for example, an analysis of population-based surveys revealed that the prevalence of epilepsy was 8.7 per 1,000 population, and the treatment gap was 77.9% [5].

Recent events such as the coronavirus disease 2019 (COVID-19) pandemic have also highlighted the importance of understanding knowledge, attitudes, and practices (KAP) toward health issues. A cross-sectional study conducted among university students in Pakistan found that despite high levels of knowledge about COVID-19, there were gaps in the implementation of preventive measures [6]. Similarly, a study conducted in Pakistan revealed that many patients had poor knowledge about epilepsy and its management [7]. Cross-sectional research in Saudi Arabia discovered that the public's understanding, views, and practices regarding the seasonal influenza vaccine were insufficient [8-10].

Therefore, it is essential to understand the KAP of individuals toward epilepsy and its management to develop effective public health strategies to reduce the treatment gap and improve the life quality of individuals with epilepsy. This paper aims to assess the literature on the knowledge and awareness of individuals toward epilepsy and its management in different populations and settings. This would help the degree of readiness of the Pakistani community to address the burden of anxiety.

Epilepsy is a highly stigmatized disorder in Pakistan, and there is a significant lack of knowledge and awareness about the disorder among the general population [9,10]. This study provides valuable insights into the KAP related to epilepsy in Pakistan and highlights the need for education and awareness campaigns to address the knowledge gaps and misconceptions related to epilepsy. It is crucial that healthcare professionals, policymakers, and the general public work together to reduce the stigma associated with epilepsy and to ensure that people with epilepsy receive the care and support they need to lead fulfilling lives.

## Materials and methods

A population-based cross-sectional design was conducted in Chakwal District, Pakistan between February 1, 2018, and June 1, 2020, to evaluate the general knowledge and attitudes of the public toward epilepsy. A non-probability convenience sampling technique was utilized to recruit participants from different socioeconomic backgrounds across Chakwal District.

All individuals aged 18 years or older with at least 12 years of education were eligible to participate in the study. Individuals who declined to participate or were unable to provide informed consent were excluded from the study.

Ethical considerations were taken into account in this community-based cross-sectional study. The study synopsis was pre-approved by the Institutional Review Board (IRB)/Ethical Review Committee (ERC) before the data collection process began. All participating individuals were narrated the purpose and nature of the study, and their informed consent was requested before any data were collected. To maintain the confidentiality and privacy of the participants, all data were collected anonymously, and no personal identifying information was recorded. Before beginning the study, the purpose and nature of the research were explained to each participant, and informed consent was obtained. Participants were informed that their participation was voluntary and that they had the right to withdraw at any time.

The study focused on several variables, such as knowledge about epilepsy (yes/no) and the percentage of people who have witnessed seizures, as well as sources of knowledge, subjective causes of epilepsy, beliefs in cure, transmission, and treatment options. Additionally, variables including age, sex, marital status, and academic status were also considered.

In this study, data acquisition was done using a previously validated structured questionnaire [11] to gather information about the general knowledge and attitudes of the public toward epilepsy, as well as its associated factors. The questionnaire was customized after a thorough examination of pertinent literature and pretested to ensure that it was clear, easy to understand, and valid. The questionnaire consisted of open-ended and closed-ended questions, and it was translated into Urdu, the national language of Pakistan, for participants who had difficulty understanding English. The questionnaire was administered face-to-face by trained research assistants who were fluent in both English and Urdu. Once the data collection process was complete, the responses were entered into a computer software program for statistical analysis.

Upon completion of data collection, responses were entered into SPSS version 21 software (IBM Corp., Armonk, NY) for statistical analysis. Descriptive statistics were used to summarize the data, with nominal and ordinal datasets presented as frequency and proportions, while continuous data were presented as mean and standard deviation (SD). All parameters were presented in tabular form.

## Results

The survey included 512 participants, and the age distribution was as follows: 18-29 years old accounted for 18% of the respondents, 30-44 years old accounted for 35%, and 45-60 years old accounted for 31%. There was a female predominance with a frequency of 312 (60.9%) (Table 1).

**Table 1:** Sociodemographic factors of study participants (n = 512)

Parameter	Frequency	Percentage (%)
Age groups		
18-29 years	131	25.6%
30-44 years	186	36.3%
45-60 years	195	38.1%
Gender		
Male	200	39.1%
Female	312	60.9%
Employment		
Unemployed	239	46.7%
Semi-skilled	206	40.2%
Unskilled	62	12.1%
Professional	5	1.0%

When asked about their sources of knowledge about epilepsy, the majority of participants (59.57%) reported learning about epilepsy from friends and relatives. A smaller percentage (18.36%) reported learning about epilepsy from schools, while another 20.31% heard about epilepsy from media and relatives (Table 2).

**Table 2:** Knowledge, attitude, and practices of epilepsy among educated individuals in Pakistan

Parameters	Frequency (N)	Percentage (%)
Knowledge of epilepsy	512	100%
Witnessed seizures	367	71.67%
Sources of knowledge		
Friends and relatives	305	59.57%
Schools	94	18.36%
Media and relatives	104	20.31%
Other	9	1.76%
Cause of epilepsy		
Psychiatric disorder	188	36.7%
Caused by Jinns	265	51.76%
Curse of God	66	12.8%
Due to ancestral sin		
Belief in cure	212	41.40%
Transmission	259	50.5%
Treatment options		
Herbs	99	19.3%
Ruqya and traditional medicine	186	36.3%
Honey during an epileptic attack	115	22.5%
Cautery	80	15.6%
No treatment		
Is epilepsy a mental illness?	310	60.5%
Is epilepsy a hereditary disease?	151	29.5%

Out of 512 patients, 60.5% of respondents believed that epilepsy was a mental illness, while 29.5% believed that it was a hereditary disease (Table 2).

## Discussion

The findings of our study suggest that in Pakistan, the primary source of knowledge about epilepsy for the general population is from friends and relatives. More than half of the participants (59.57%) reported learning about epilepsy from people they know. This could be attributed to the cultural norms and practices in Pakistan, where the community plays an essential role in an individual's life, and information is often shared through personal networks. Similar findings have been presented in a study based in Saudi Arabia [11] in which approximately two-thirds of the participants indicated that their primary source of knowledge regarding epilepsy was acquired through informal channels such as friends and relatives. Previous studies conducted in Asia have examined the KAP regarding epilepsy among specific groups, including school teachers, patients, and healthcare providers, in comparison to the general public. However, a comprehensive investigation into the sources of knowledge has not been conducted in Asia, which limits our ability to make direct comparisons in terms of quantity or prevalence within Asia.

Only a small proportion of participants (18.36%) reported learning about epilepsy from schools. This indicates a potential gap in the education system regarding epilepsy, which could impact the public's understanding and attitudes toward the condition. Efforts to educate students and teachers about epilepsy could improve the awareness of the condition in the general population as demonstrated by existing scientific literature.

The media and relatives were also reported as sources of knowledge about epilepsy by a fifth of the participants (20.31%). This finding highlights the importance of using various channels, including mass media, to impart awareness about epilepsy to the masses. Additionally, involving relatives in educational initiatives could help increase their understanding of epilepsy and promote more supportive attitudes toward people living with the condition.

The finding that the majority of participants learned about epilepsy from friends and relatives emphasizes the importance of social networks and peer education in raising awareness about epilepsy. Similarly, a study conducted in Ethiopia found that public knowledge and attitudes toward epilepsy were poor and that there was a significant gap in understanding of the condition among the community [12]. Furthermore, a study in Northwest Ethiopia found that there was a huge gap in knowledge and attitudes toward epilepsy among the community [13].

The current study found that the majority of participants believed that epilepsy is caused by possession by Jinns or a curse of God, highlighting the role of religious and supernatural beliefs in shaping attitudes toward the condition. This research is in line with previous studies that found that religion and spirituality play a significant role in shaping beliefs about epilepsy, particularly in countries where traditional and supernatural beliefs are prevalent [14,15].

The finding that traditional medicine and Ruqya were the preferred choices of treatment for most participants highlights the need for culturally sensitive approaches to epilepsy management. Similarly, a study conducted in Saudi Arabia found that traditional medicine was commonly used to treat epilepsy [16]. Another study found that the overall knowledge of seizure first aid among the participants was poor, with only 33.9% of participants scoring above 50% on the knowledge test. Furthermore, 45.5% of participants reported that they would not intervene during a seizure, highlighting the need for education and training on seizure management [17].

Overall, this study highlights the need for targeted educational initiatives and awareness campaigns to increase knowledge about epilepsy in Pakistan. Such efforts could help reduce the stigma and discrimination faced by people living with epilepsy and improve their quality of life [18,19].

There are a few limitations in the study. Firstly, the study used a randomized sampling technique; however, it was limited to individuals aged 18 years or older with at least 12 years of education. This restriction may have led to a selection bias that could restrict the generalizability of the findings to the general population. Secondly, there is a possibility of social desirability bias in the present research, where participants may have provided answers that they deemed socially acceptable rather than their true knowledge or attitudes toward epilepsy. Thirdly, the study relied on self-reported data, which is subjected to recall bias, as participants may have forgotten important details or experiences related to epilepsy. Finally, the study was conducted only in the Chakwal District, which may not be representative of the entire population of Pakistan. The findings may not be generalizable to other regions or settings within Pakistan.

Therefore, we recommend that further studies should be conducted to overcome the limitations by broadening the sampling strategy, using a blinded sampling technique, and use of validated and reliable measures.

## Conclusions

The present study highlights a significant lack of understanding and knowledge about epilepsy among the general population in Pakistan. Myths and misconceptions about epilepsy being a mental illness and hereditary disease were prevalent among the participants, indicating a need for targeted education and awareness campaigns to burst such myths. Additionally, the majority of participants received knowledge of epilepsy from their friends or close relatives, highlighting the importance of peer education and social networks in raising awareness about the condition.

The findings of this study suggest the need for culturally sensitive approaches to epilepsy management, including the provision of accurate and accessible information about epilepsy, community-based awareness campaigns, and the involvement of religious and community leaders in raising awareness about the condition.
